# Post-Hospital Discharge Strategy for COVID-19 Treatment and Control: Focus on Fangcang Hospitals

**DOI:** 10.1017/dmp.2021.83

**Published:** 2021-03-26

**Authors:** Saeed Kassaeian, Ali Gohari, Gholamreza Masoumi, Zohreh Ghomian, Arezoo Dehghani

**Affiliations:** 1 Department of Community Medicine, Semnan University of Medical Sciences, Semnan, Iran; 2 Department of Internal Medicine, Semnan University of Medical Sciences, Semnan, Iran; 3 Health in Emergencies and Disasters Department, School of Health Management and Information Services, Iran University of Medical Sciences, Tehran, Iran; 4 Emergency Medicine, Emergency Management Research Center, Iran University of Medical Sciences, Tehran, Iran; 5 Health in Emergency and Disaster Research Center, University of Social Welfare and Rehabilitation, Tehran, Iran; 6 Department of Health in Emergencies and Disasters, School of Public Health and Safety, Shahid Beheshti University of Medical Sciences, Tehran, Iran; 7 Department of Students’ Scientific Society, School of Public Health and Safety, Shahid Beheshti University of Medical Sciences, Tehran, Iran

**Keywords:** COVID-19, Fangcang hospital, pandemic, emerging diseases, disasters

## Abstract

**Objective:**

One of the concerns of health managers in Iran in case COVID-19 reached a new peak is a shortage of hospital beds. In response, the country designed and created intermediate treatment centers, known as *fangcang* hospitals, which are prepared quickly at low cost and with high capacity. The aim of this study is to provide health managers with an effective post-hospital discharge strategy for COVID-19 patients.

**Method:**

The study was conducted from April 2020 to June 2020, with a narrative case study design. Setting up a fangcang hospital was based on a narrative analysis of 2 in-depth interviews with 4 fangcang hospital managers in Iran, a field visit of these places, and a review of their protocols and guidelines.

**Result:**

The patient flow for screening, treatment, and follow-up includes the following: Patients will be hospitalized if their symptoms are severe. If they are infected with mild symptoms, they will be referred to a fangcang hospital and admitted there if necessary, to prevent further spread of the disease. Patients will be monitored regularly and treated with routine health services. At the end of the 14-day quarantine period, patients approved for discharge are sent home.

**Conclusion:**

Traditional hospitals and fangcang hospitals are working together under the supervision of the Iran University of Medical Sciences. Our experience can serve as guidance for other clinics and recovery shelters. Having guidelines in place assists health care workers and managers in responding quickly to patients' needs during times of a disaster.

Coronavirus disease (COVID-19) is a new illness that affects the human respiratory system and is caused by severe acute respiratory syndrome coronavirus 2 (SARS-CoV-2), a virus that belongs to the family of coronaviruses.^1^ COVID-19, the disease, was discovered in December 2019. It emerged in the city of Wuhan, China, and has since become a pandemic. The health system during the COVID-19 outbreak has focused on identifying, treating, and isolating patients; tracing and quarantining; promoting risk perception; and promoting hygiene among the general public.^2^

Up until February 7, 2021, there were almost 106 million reported cases, and, so far, over 2 million people globally have died.^3^ Iran is currently experiencing ongoing, widespread transmission of COVID-19 with almost 1.5 million cases and 58 469 deaths.^4^

One of the concerns of health managers is that, if COVID-19 was to have a new peak, the country would have a shortage of hospital spaces and beds. In this regard, one of the great measures of the Armed Forces and non-governmental organizations was to design and create post-hospital spaces, known as a *fangcang hospital*. A fangcang hospital is an intermediate treatment center that is prepared at low cost, quickly, and with high capacity. These centers provide primary care services.^5^ More than 27 000 beds were provided to the health system in more than 300 locations with good logistical, service, pharmaceutical, and therapeutic measures, as well as more than 3000 nursing volunteers and more than 2000 social worker volunteers worked at these fangcang hospitals.^6^

Due to the nature of the disease and the number of patients, the guidelines of the COVID-19 Patient Care Unit after discharge from the hospital (recovery center) was announced to all medical universities in Iran on March 3, 2020 (2 weeks after the first case was identified). The second edition of the guidelines was issued on March 30 (2 weeks after the first version was announced) as a guideline for the COVID-19 Outpatient Care Unit.

Depending on the nature of the disease, it can take up to 14 days for the onset of symptoms from the time of exposure, how to refer the patient, the time of referral, the place of referral, and some social and economic characteristics of the patient, the decision about admission to a regular hospital or fangcang hospital, hospital discharge, and departure to home or fangcang hospital is different.

People can be admitted to a fangcang hospital, who have a positive polymerase chain reaction (PCR) test result or a CT scan, or who had been hospitalized with a diagnosis of COVID-19 and was discharged after the general condition improvement and before the end of 14 days from the onset of symptoms. The same procedure can be followed for people who have been diagnosed and tested positive at a comprehensive health care center.

Traditional hospitals and fangcang hospitals are working together under the supervision of the University of Medical Sciences. The Ministry of Health adjusted referral clinics for outpatients. People with symptoms of COVID-19 diseases went to the hospital, and some others went to referral clinics. Patients will be hospitalized if their symptoms are severe. If they are infected with mild symptoms, they will be referred to a fangcang hospital if they have personal satisfaction with hospitalization there.

The family members are monitored through tests and CT scans, and if the results are positive, the infected person will be hospitalized. If the results are not positive, the health status of the family members will be monitored daily for 14 days.

If they are not satisfied with hospitalization in the fangcang hospital but have the following conditions, they must be admitted there in order to prevent further spread of the disease (Figure 1).

**Figure 1.**
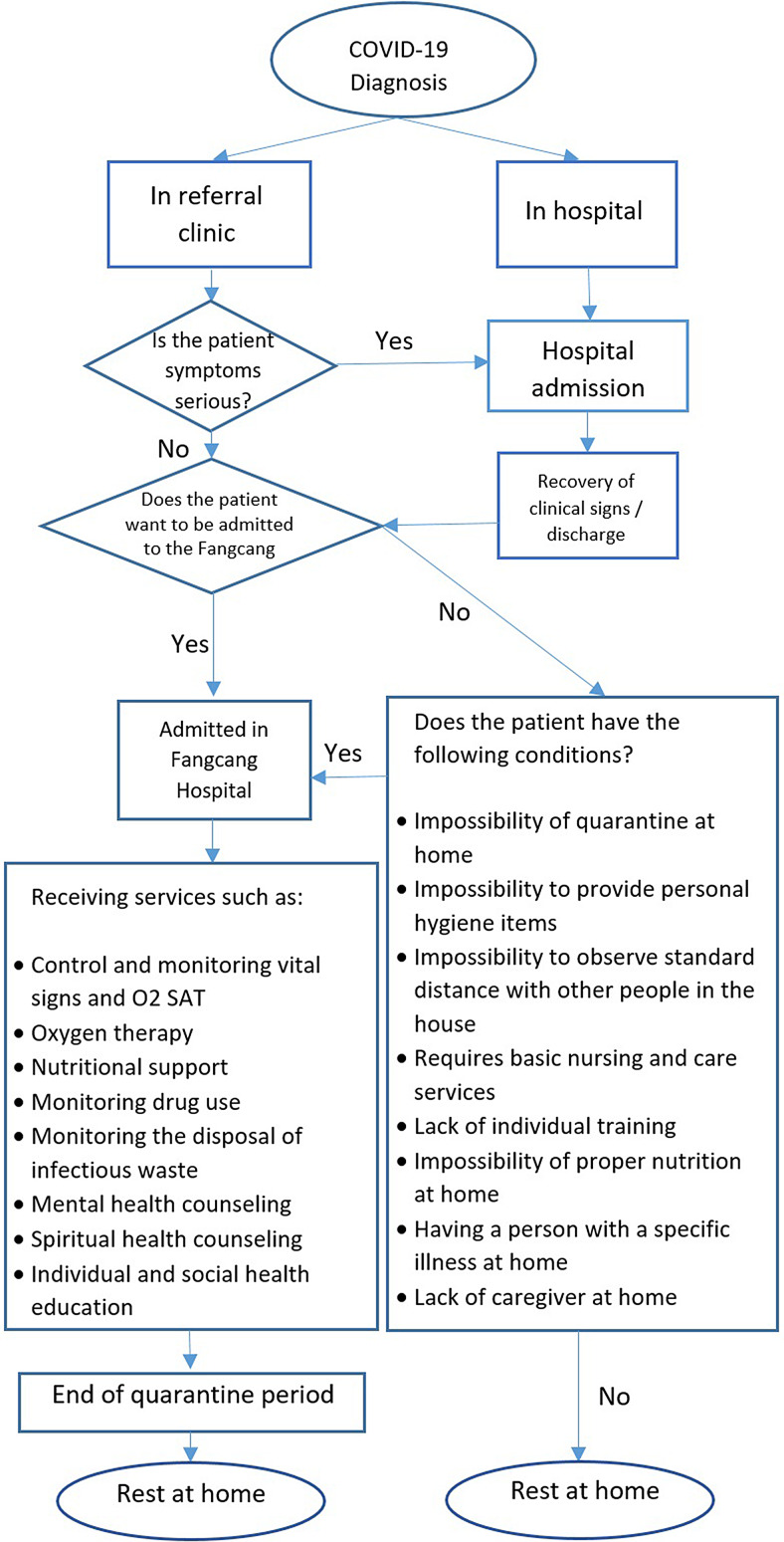
Flow chart for patient screening, treatment and follow-up of COVID-19 in fangcang Hospital in IRAN.

Patients who are discharged from the hospital should pass the same process, follow up, and continue to the quarantine period in the fangcang hospital.

The University of Medical Sciences has created a law that patients with COVID-19 who need to be admitted to the fangcang hospital will be transported there from both the hospital and referral clinic with the ambulance. The quarantine period is calculated from the onset of symptoms through 14 days.

Patients with mild to moderate physical symptoms are constantly monitored and provided with health services, such as control and monitoring of vital signs and O_2_ saturation, oxygen therapy, nutritional support, monitoring of drug use, monitoring the disposal of infected waste, and so forth.

Due to mental challenges and conditions of the hospitalized patients, the lack of visits, the distance from their families, and the long hospital stays, a mental health counselor and a spiritual health counselor are provided in the fangcang hospital.

The incubation period for COVID-19, which is the time between exposure to the virus (becoming infected) and symptom onset, is on average 5–6 days but can take up to 14 days for symptoms to show^7^; therefore, it is possible for others to be infected within this time period. Arrangements are made for families of patients hospitalized in a fangcang hospital.

After the person is diagnosed with the disease and transferred to a hospital or Fangcang hospital, the patient’s home will be disinfected.

We hope our experience will serve as guidance for other clinics, fangcang hospitals, and recovery shelters. This flowchart can be used in the future, in the context of pandemics, epidemics, and the spreading of communicable diseases.
